# Methylphenidate enhances neuronal differentiation and reduces proliferation concomitant to activation of Wnt signal transduction pathways

**DOI:** 10.1038/s41398-018-0096-8

**Published:** 2018-03-01

**Authors:** Edna Grünblatt, Jasmin Bartl, Susanne Walitza

**Affiliations:** 1 Department of Child and Adolescent Psychiatry and Psychotherapy, University Hospital of Psychiatry Zurich, University of Zurich, Zürich, Switzerland; 2 Neuroscience Center Zurich, University of Zurich and the ETH Zurich, Zürich, Switzerland; 3 Zurich Center for Integrative Human Physiology, University of Zurich, Zürich, Switzerland; 4 Department of Pediatric Oncology, Hematology, and Clinical Immunology, University Hospital Düsseldorf, Düsseldorf, Germany

## Abstract

Methylphenidate (Ritalin) is the most commonly prescribed drug in the treatment of attention-deficit hyperactivity disorder. It is suggested that in vivo, methylphenidate treatment supports cortical maturation, however, the molecular and cellular mechanisms are not well understood. This study aimed to explore the potential effect of methylphenidate on cell proliferation and maturation in various cellular models, hypothesizing its interaction with the Wnt-signaling. The termination of cell proliferation concomitant to neuronal maturation following methylphenidate treatment was observed in all of the cell-models tested: murine neural stem-, rat PC12- and the human SH-SY5Y-cells. Inhibition of Wnt-signaling in SH-SY5Y cells with Dkk1 30 min before methylphenidate treatment suppressed neuronal differentiation but enhanced proliferation. The possible involvement of the dopamine-transporter in cell differentiation was discounted following the observation of opposing results after GBR-12909 treatment. Moreover, Wnt-activation via methylphenidate was confirmed in Wnt-luciferase-reporter assay. These findings reveal a new mechanism of action of methylphenidate that might explain long-term effects.

## Introduction

The neurodevelopmental disorder attention-deficit hyperactivity disorder (ADHD) is one of the most common psychiatric and behavioral disorders in children and adolescents, often persisting into adulthood^1^. Delayed cerebral cortex maturation (thickness and surface area), in particular in frontal regions known to be important in the control of cognitive processes concomitant to alterations in basal ganglia formations, were reported in children and adolescents with ADHD^2,3^. These have also been linked to adult ADHD^4^. Moreover, ventral and dorsal striatal surface reduction, associated with reward processing, executive function and motor planning, respectively^5,6^ have also been observed in ADHD. Psychostimulants, in particular methylphenidate (MPH) and amphetamine, provide an effective treatment for ADHD^7^, although their effects on brain development, maturation and its safety have been discussed controversially^8–11^. Nevertheless, several studies point to the beneficial effects of psychostimulant treatment on brain function and structure in ADHD^6,9–11^. So far it is known that MPH binds with high affinity to the dopamine transporter, and with lower affinity to the norepinephrine transporter and serotonin transporter, and inhibits the transport of synaptic monoamines back into the neuron^12^. However, given the extent of MPH prescription^13^, and the levels of misuse amongst youth and young adults^14^, particularly at sensitive developmental stages in regard to brain maturation, concerns over adverse effects (e.g., neurogenesis, neuronal development, maturation, receptor densities or connectivity) have been raised. Few studies have looked into neurodevelopmental effects of MPH treatment, in vitro and in vivo. In vivo, several studies have observed that MPH treatment was associated with neurogenesis and cell differentiation in rodents^15,16^. Recently, 2 and 5 mg/kg (oral) MPH treatment in adolescent rats over a five-week period reportedly increased hippocampal neurogenesis and inward shape deformations. This was not observed in adult rats with the same treatment^17^. An in vitro study of neuronal maturation following MPH treatment (1–100 nM, doses correlating to oral intake of 1 mg/kg MPH in patients’ serum^18,19^) in murine neural stem cells (mNSC) found that MPH enhanced cell differentiation, but inhibited cell proliferation^20^. However, the cellular and molecular mechanism affected by MPH treatment is not yet fully understood.

Therefore, the current study aims to extend our previous mNSC pilot study^20^ by using three cell culture models: mNSC, rat pheochromocytoma-12 (PC12) and the human neuroblastoma SH-SY5Y cells. Moreover, following the observed differentiation effect of MPH on the cells, we hypothesized the possible involvement of the canonical Wnt-signaling pathway^21–23^. Our hypothesis is based on the fact that Wnt-signaling plays a role in several cellular and physiological processes, regulating cell proliferation, differentiation, migration, and patterning during embryonic development, and tissue homeostasis in adults, but is specifically needed in the maturation and differentiation of NSC^24^. Two approaches will be used to test this hypothesis. First, a pharmacological approach in which we will compare the effects of treating the human SH-SY5Y cells with MPH to those treated with either R-spondin 1 (Wnt activator), GBR-12909 (selective dopamine transporter inhibitor), or by blocking the Wnt-signaling with Dickkopf-1 (Dkk1; antagonistic inhibitor of the Wnt-signaling, binds to low-density lipoprotein receptor-related protein 5/6; LRP5/6) 30 min before MPH treatment. In the second approach, we will use a cell-based luciferase assay in mouse fibroblast cells engineered with the firefly luciferase reported gene under the control of Wnt-responsive promoters (TCF/LEF), enabling us to measure Wnt/β-catenin-activity after pharmacological treatment with MPH.

## Material and methods

### Preparation of compound solution

Specification of compound solution preparation is described under Supplementary Material And Methods in details.

### Murine neuronal stem cell (mNSC) sphere monolayer culturing and differentiation study

**Fig. 1:**
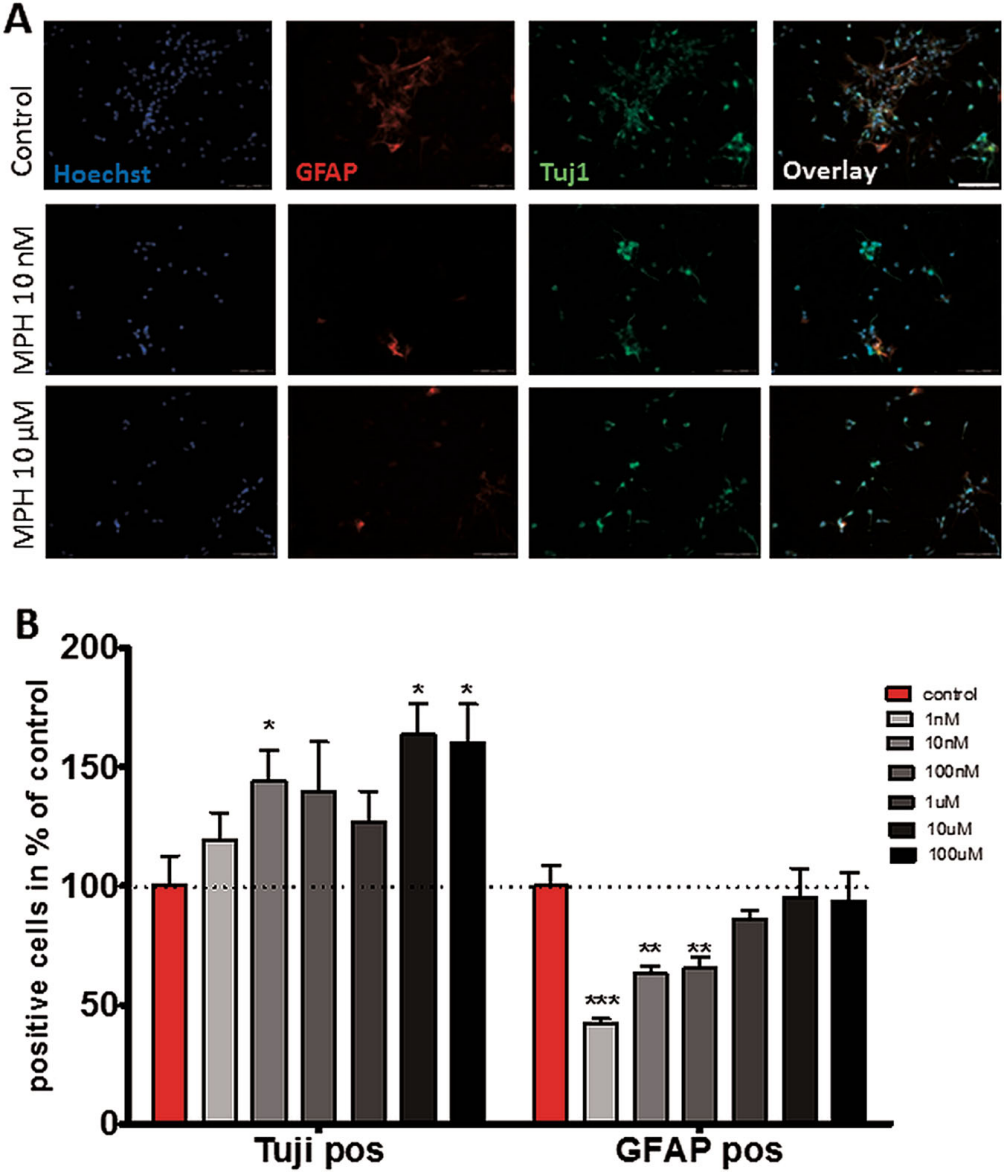
Effect of MPH on embryonic mNSC differentiation into astrocyte (GFAP^**+**^ cells) and neurons (Tuj1^**+**^ cells). **a** Representative immunocytochemistry staining (control, MPH 10 nM and 10 μM; see Supplementary Fig. S1 for all MPH doses) of embryonic mNSC against GFAP (red), Tuj 1 (green) and for total cell count using Hoechst 33258 staining (blue). Scale bar = 50 µm. **b** MPH treatment induced differentiation of mNSC into Tuj1 positive neurons showing significance starting with 10 nM MPH treatment, while suppressing differentiation into GFAP-positive astrocytes. Results presented as percent of control treatment showing mean value, and the s.e.m. Kruskal–Wallis followed by Mann–Whitney Test *p < 0.05 vs. control, **p < 0.01 vs. control, ***p < 0.001 vs. control; *n* = 5 repeats

mNSCs were derived from the hippocampus tissue of albino mouse (Charles River, Switzerland) embryos on embryonic day 15 (E15). All animal procedures were performed in accordance with National Institute of Health, and the Cantonal Veterinary office Zurich for the care and use of laboratory animals’ guidelines (Ethic permission 223/2014). Cells were cultured in a defined medium (NeuroCult Proliferation Kit (Mouse); Stemcell, Germany) for 6 days at 37 °C and 5% CO_2_. After 6 days, the grown NSC spheres were harvested and prepared for differentiation. mNSC spheres were collected and plated on a 30 μg/ml poly-l-lysine (Sigma aldrich, Switzerland) and 20 μg/ml laminine (Invitrogen, Switzerland) coated 8-well glass cover-slip and incubated for another four days creating a monolayer (see example Fig. 1a). Each well had a final concentration of 22.8 × 10^4^ cells/ml cultured in NeuroCult Differentiation Kit (Mouse) (Stemcell, Germany). The cells were treated directly after seeding to the cover-slip using different concentration (1 nM, up to 100 μM) of MPH or water as control. On the last day, cells were fixed with 4% paraformaldehyde for 20 min at room temperature and stained for glial fibrillary acidic protein (GFAP; 1:200, Sigma aldrich, USA, Cat-No: C4546), β-tubulin III (Tuj 1; 1:300, Sigma aldrich, USA, Cat-No: T3952), and Hoechst 33258 staining (1:100; Invitrogen, Cat-No: H3569) (for detailed Immunocytochemistry procedure see supplementary material and methods).

#### Statistical analysis

The staining’s were analyzed for the cell count of astrocytes (GFAP-positive cells) and immature neurons (Tuj 1 positive cells) in comparison to the total number of cells (Hoechst positive cells) using the Kruskal–Wallis followed by Mann–Whitney Test. In order to present results in comparison to the untreated cells, percentages were calculated for each treatment dose, setting the control as 100%. Analysis was conducted using SPSS version 22 software program (IBM, USA). A *p*-value < 0.05 was set as significant. There were at least five independent experiments, and five pictures per well (treatment) were analyzed.

### Neuronal cell line culture

For the standard culturing and differentiation procedure of human neuroblastoma SH-SY5Y cells (European Collection of Authenticated Cell Cultures (ECACC), UK) and rat pheochromocytoma (PC12) cells (ECACC, UK) see supplementary material and methods.

#### Treatment of SH-SY5Y and PC12 cells

**Fig. 2:**
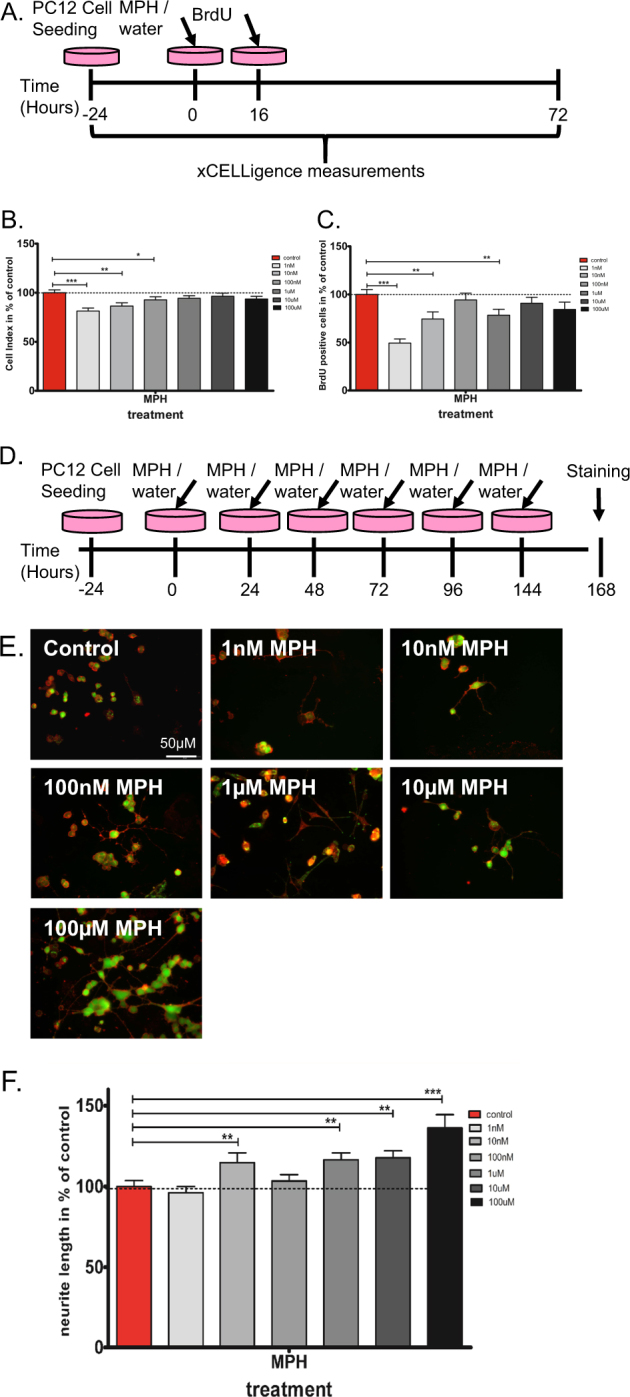
Influence of MPH treatment on PC12 cells proliferation and differentiation. Proliferation: (**a**) the time line of cell culturing, treatment and proliferation measurements. **b** Mean cell index (impedance measured by xCELLigence) values as % of control ± s.e.m. at 16 h after MPH treatment (1 nM–100 µM) showing significant decrease in cell proliferation. **c** Mean of BrdU positive cells as % of control ± s.e.m. at 16 h after MPH treatment (1 nM–100 µM) resulted in a significant decrease in cell proliferation. Mann–Whitney *p < 0.05; **p < 0.01; ***p < 0.005. Differentiation: (**d**) the time line of cell culturing, treatment and neurite outgrowth determination via ‘Neurite outgrowth staining kit’ (Thermo Fisher) for differentiation evaluation. **e** Representative neurite outgrowth staining of vehicle and MPH (1 nM–100 µM) treated PC12 cells. Scale bar = 50 µM. **f** Neurite outgrowth was significantly increased after 10 nM up to 100 μM MPH treatment. Mann–Whitney test **p < 0.05 and ***p < 0.01 vs. control. *n* = 4–5 independent experiments with 4–6 internal replications

In the proliferating experiment, PC12 cells were treated once with either MPH or water (Fig. 2a). In the differentiation experiment, PC12 cells were treated every 24 h with either MPH or water and the morphological classification of cell differentiation was done after 6 days of culturing in differentiation medium (DM), consisting of a normal growth medium supplemented with 10 μM retinoic acid (Sigma Aldrich, Switzerland) and 50 ng/ml nerve growth factor (Sigma Aldrich, Switzerland; Fig. 2d).

**Fig. 3:**
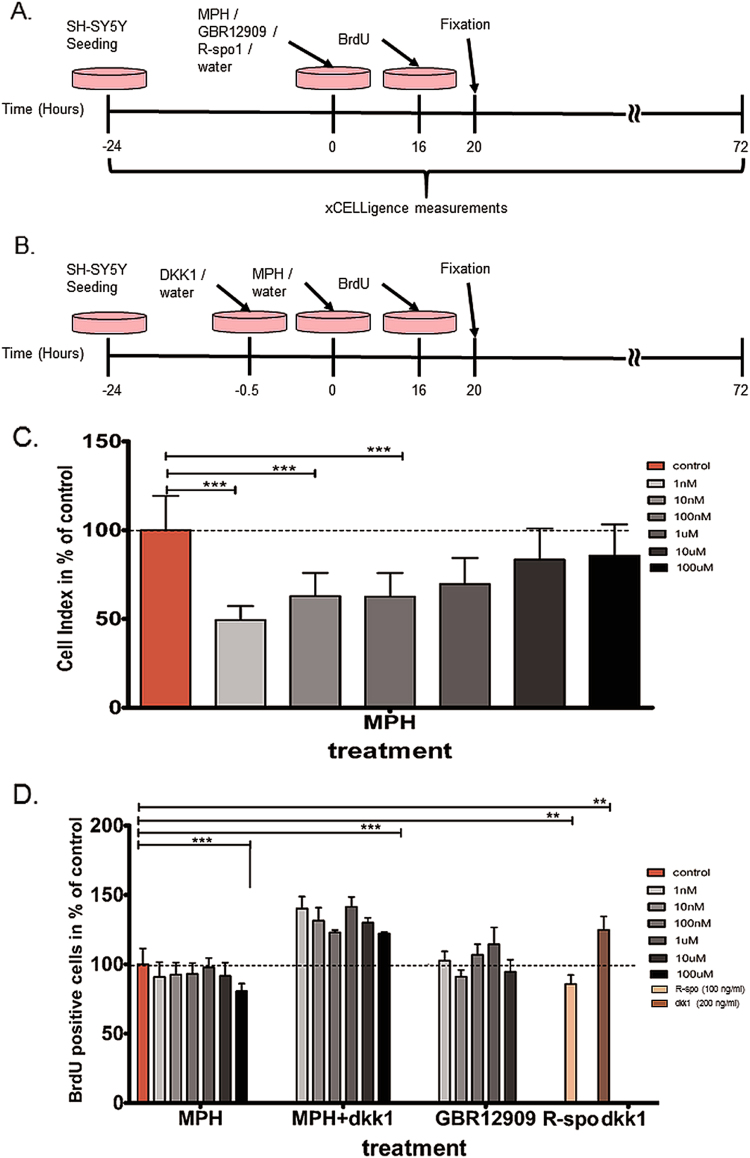
Influence of MPH treatment on SH-SY5Y cells proliferation and the involvement of Wnt-signaling. **a** The time line of cell culturing, treatment with either MPH (1 nM–100 μM), GBR12909 (selective dopamine transporter inhibitor; 1 nM–100 μM) or R-spondin 1 (R-spo1; Wnt activator, 100 ng/ml) and proliferation measurements. **b** The time line of cell culturing, inhibiting Wnt-signaling by treating cells with Dkk1 (LRP inhibitor; 200 ng/ml) half an hour before MPH treatment and proliferation measurements. **c** Mean cell index (impedance measured by xCELLigence) values as % of control ± s.e.m. at 16 h after MPH treatment (1 nM–100 µM) showing significant decrease in cell proliferation. **d** Mean of BrdU positive cells as percentage of control ± s.e.m. at 16 h after MPH treatment (1 nM–100 µM) resulted in lower cell proliferation. Blocking Wnt-signaling using Dkk1 enhanced significantly cell proliferation. Similarly, inhibiting Wnt-signaling with Dkk1 30 min before MPH treatment resulted in increased proliferation. As expected, activation of Wnt-signaling by R-spondin 1 reduced proliferation of the cells. Moreover, the selective dopamine transporter inhibitor GBR12909 did not affect SH-SY5Y cell proliferation. *n* = 5 independent experiments; *N* = 18302 counted cells; Mann–Whitney test, *p < 0.05, **p < 0.01, ***p < 0.001

**Fig. 4:**
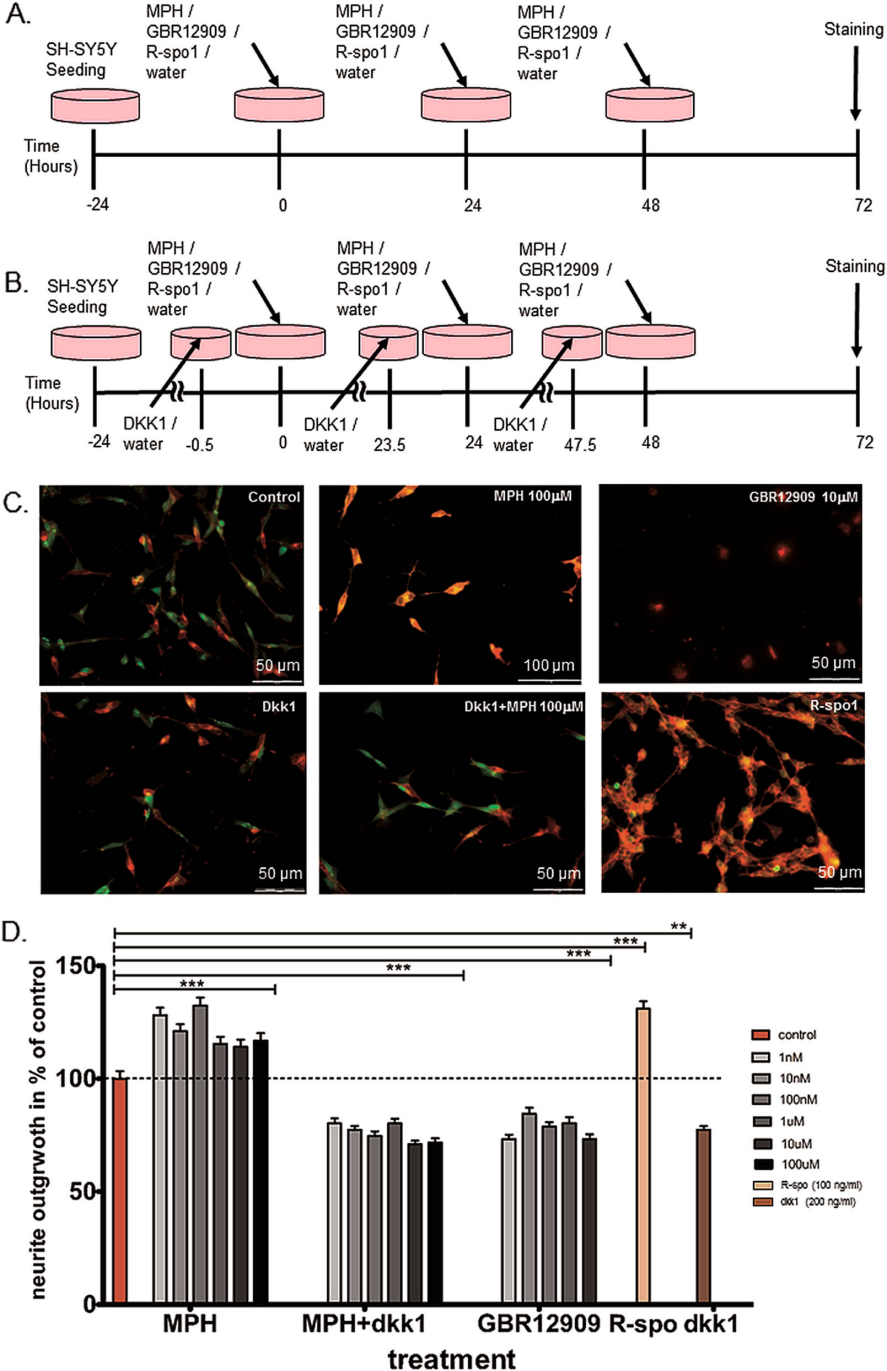
Influence of MPH treatment on SH-SY5Y cells differentiation and the involvement of Wnt-signaling. **a** The time line of cell culturing, treatment with either MPH (1 nM–100 μM), GBR12909 (selective dopamine transporter inhibitor; 1 nM–100 μM) or R-spondin 1 (R-spo1; Wnt activator, 100 ng/ml) and neurite outgrowth determination via ‘Neurite outgrowth staining kit’ (Thermo Fisher) for differentiation evaluation. **b** The time line of cell culturing, inhibiting Wnt-signaling by treating cells with Dkk1 (LRP inhibitor; 200 ng/ml) half an hour before MPH treatment and differentiation evaluation. **c** Representative neurite outgrowth staining of vehicle, MPH (100 µM), GBR12909 (10 µM), Dkk1 (200 ng/ml), Dkk1 + MPH (100 µM) and R-spondin 1 (100 ng/ml) treated SH-SY5Y cells. Scale bar = 50–100 µM. **d** Neurite outgrowth was significantly increased after MPH treatment, which was inhibited significantly by Dkk1 treatment. Similar to MPH, R-spondin 1 treatment increased significantly cell differentiation. However, the selective dopamine transporter inhibitor, GBR12909, caused a significant decrease in SH-SY5Y cell differentiation, while the higher dose of 100 µM induced cell death. *n* = 3–5 independent experiment; *N* = 2172 measured cells; Mann–Whitney test, **p < 0.05, ***p < 0.001 vs. control

In the proliferating experiments, SH-SY5Y cells were treated once with either MPH, GBR12909 (cat. no. D052; Sigma Aldrich, Switzerland), R-spondin 1 (cat. no. SRP6487; Sigma Aldrich, Switzerland), compared to vehicle water or DMSO. For Wnt-signaling pathway inhibition, Dkk-1 (cat. no. SRP3258; Sigma Aldrich, Switzerland) was added 30 min before cells were treated with MPH (Fig. 3a, b). In the differentiation experiment, SH-SY5Y cells were treated every 24 h with different doses of either MPH, GBR12909, R-spondin 1, compared to vehicle water or DMSO. While in the Wnt-signaling pathway inhibition, Dkk-1 was added 30 min before cells were treated with MPH. The morphological classification of cell differentiation was conducted after 3 days of culturing in DM (Fig. 4a, b).

#### xCELLigence analysis for cell proliferation or cytotoxicit***y***

Cell viability and proliferation after acute treatment with MPH was monitored using the real-time xCELLigence analyzer from Roche. 96-well E-plates (ACEA, USA) were coated using 40 µl/well collagen-I solution (ScienCell, USA) for both cell types. The background cell impedance of the pure growth medium without cells was measured, before SH-SY5Y cells were seeded at a concentration of 5×10^4^ cells/well and PC12 at 1×10^4^cells/well. The baseline cell index (CI) was measured every 15 min for 24 h. Afterwards the cells were treated with different doses of MPH, whereas CI was measured continuously every minute for two hours and every 15 min for another 48 h.

##### Statistical analysis of xCELLigenece results

Raw data from the xCELLigence system presented as CI values were used for the statistical analysis with the statistical software program StatView 5.0 (SAS Institute Inc.Cary, NC, USA). Determination of the first significant time point in which MPH alters cell proliferation in comparison with untreated cells was conducted using self-written MATLAB programs in which all impedance measures with time and dose were evaluated (Supplementary Fig. S2 B and C). For this, the CI data were exported from the xCELLigence software into an excel file followed by coma separated value file. For each time point measurement, outliers were detected and removed (CI values more than two s.d., but only if it was more than one s.d. away from the plate mean at this time point). Comparison was carried out for each measured time point by calculating mean, s.d. and number of wells used to calculate the two values for the two replicate groups. The Welch’s *t-*test was applied. The *p*-value was plotted semi logarithmic and with inverted axis against the time scale, thus the more significant a p-value, the higher the peak on the plot. *p*-value < 0.01 were set as significant (Supplementary Fig. S2C). The demonstrated values were calculated in percent relative to control levels (100%; control was water).

#### BrdU Incorporation

The SH-SY5Y cells were seeded into poly-d-lysine/laminin coated eight-well slides at a density of 3×10^4^ cells/well and the PC12 cells were seeded at the same density into Collagen I coated eight-well slides. The cells were incubated at 37 °C in a humidified atmosphere of 5% CO_2_ for 24 h. SH-SY5Y cells were treated with different doses of MPH (1 nM–100 µM), GBR12909 (1 nM–10 µM), R-Spondin1 (100 ng/ml), dkk1 (200 ng/ml) alone, or 30 min before MPH was added, (vehicle was water or DMSO) and incubated for 16 h. This time point of assessment was determined according to the xCELLigence results (see section ‘xCELLigence analysis for cell proliferation or cytotoxicity’). PC12 cells were treated with either MPH or water followed by incubation for 16 h. After incubation 10 μM of the thymidine-analog 5-bromodeoxyuridine (BrdU) was added to the cells and incubated for 4 h at 37 °C. Cells were fixed with 4% ice-cold paraformaldehyde (SantaCruz Biotechnologies, Switzerland) followed by standard procedure of staining (see Supplementary Material And Methods). Pictures were taken at ×20 magnification under the inverted microscope (Olympus IX81, Germany) with DP72 Digital camera. Cells were counted using the xcellence software (Olympus, Germany).

#### Cytotoxicity and apoptosis

ApoTox-Glo Triplex Assay and CellTox Green Cytotoxicity Assay from Promega (Switzerland) were used to determine the possible toxic effect of MPH on PC12 cells. For testing the cytotoxicity and apoptosis effect, PC12 cells were seeded at 10^4^ cells/well onto Collagen I treated black 96-well plates and were cultivated for 24 h at 37 °C in 5% CO_2_. Afterwards cells were treated with different concentration of MPH and reaction was measured 24 h later. The assays were performed according to the manufacturer´s protocol.

#### Measurement and quantification of cell differentiation

To determine neurite outgrowth, which is a hallmark of differentiated cells, of PC12 and SH-SY5Y cells the Neurite Outgrowth Kit purchased from Thermo Fisher Scientific (Switzerland) was used to stain the cell processes. The manufacturers protocol was slightly altered by substituting PBS with Opti-MEM (Thermo Fisher Scientific, Switzerland), since a loss of cells and breaking of longer neurites was observed when strictly adhering to the protocol. After aspiring the medium, the cells were stained with a working stain solution (3 μl of cell membrane indicator and 3 μl cell viability indicator in 3 ml Opti-MEM) for 15 min. The stain solution was then replaced by a background suppressor solution and pictures were taken on the Olympus DP72 microscope, using 20 × magnification. Per experiment, five representative images per condition containing at least five neurons were analyzed using the xcellence software (Olympus, Germany). To determine the differentiation state the ratio of neurite length to diameter of soma was taken as a reference. Astrocytes, glia cells and other cell types, which can still appear in cell culture, were not considered for analysis.

#### Statistical analysis

Statistical analysis was done using Prism (Graphpad Software, version 6.0). The statistical analysis of xCELLigence was done via StatView and MATLAB. Results are given as mean ± s.e.m. Analysis was conducted using Mann–Whitney test with *p* < 0.05 set as significant. For proliferation analysis over 18,000 cells were calculated and for differentiation study over 2100 cells. All experiments were repeated at least three times independently.

### Activity detection of canonical Wnt-signaling with luciferase Wnt reporter assay

To confirm our pharmacological analysis of the Wnt-signaling activation by MPH and R-Spondin1 against Wnt-signaling inhibition via Dkk1, we used the Leading Light Wnt Reporter Assay (Enzo Life Sciences, Lausen, Switzerland). The cell-based luciferase activity assay was conducted with some modifications to the manufacturer protocol (Supplementary Material And Methods). Following, Wnt agonist activity was run by adding to the cells either 5 µl R-Spondin1 100–1000 ng/ml (Sigma, Switzerland), Wnt3a 5–200 ng/ml (abcam, Switzerland), LiCl 5–40 mM (Sigma, Switzerland), or MPH 1 nM up to 1 mM (all diluted in H_2_O). Water was used as a negative control. Wnt antagonist activity test was conducted by adding to the cells already treated with Wnt3a 100 ng/ml the potential antagonist Dkk-1 20-200 ng/ml (5 µl; Enzo Life Science, Switzerland), or 5 µl MPH 1 nM up to 100 mM. Cells were incubated at least 12 h, washed with PBS, followed by 15 min incubation with lysis buffer (Biotium, USA). Lysates were transferred into white microplate (Berthold, Germany) and mixed with luciferin assay buffer (Biotium, USA). Chemiluminescence reaction was measured immediately using the Mithras LB943 (Berthold, Germany) resulting in relative luciferase unite (RLU) measures. All experiments were conducted with internal and external replications of 4–38. Regression was conducted using the exponential regression equation.

### Sclerostin-LRP interaction assay

LRP might also be involved in MPH activation of Wnt-pathway, since activation of Wnt occurs via binding of Wnt-ligands to Frizzled (Fz) and LRP receptor, triggering the canonical Wnt-signaling cascade^25^. In the regulation of bone formation, sclerostin, encoded by the SOST gene, can bind to LRP5/6, causing inhibition of the canonical Wnt signaling^26^. To evaluate, whether MPH interacts with the sclerostin-LRP binding, the Leading Light Sclerostin Interaction Screening (Enzo Life Sciences, Lausen, Switzerland) in a 96-well system was used, following manufacturer manual (Z′-factor_reproducibility_ = 0.871). Fifty microliter MPH starting from 1 nM up to 1 mM all diluted in Assay Buffer (Enzo Life Sciences, Lausen, Switzerland) containing LRP-AP fusion protein, were added to the sclerostin pre-coated plate. As positive control for binding activation, only Assay buffer containing LRP-AP fusion protein was added. Each concentration was tested in 6-plicates. As negative control the sclerostin binding inhibitor Acid Green (Enzo Life Sciences, Lausen, Switzerland) and as background control Assay Buffer without LRP-AP was used. After incubation of two hours, wells were washed 4× followed by the addition of 50 µl AP substrate reagent (Enzo) per well. After 25 min of incubation, chemiluminescence reaction was measured using the Mithras LB943 (Berthold, Germany) resulting in RLU measures. Statistical analysis was done using StatView (v. 5.0, SAS Institute Inc.). Results are given as mean RLU ± s.e.m. Analysis was conducted using Kruskal–Wallis followed by Mann–Whitney test with *p* < 0.05 set as significant.

### Gene expression omnibus (GEO) search for data sets of MPH treatment influence on transcriptomic profiling and enrichment analysis

A search of data sets under GEO was conducted for gene expression profiling results after MPH treatment conducted in mammals with the keyword ‘methylphenidate’ and limiting the search to the ‘expression profiling by array’ study type. Three data sets could be found (excluding one dealing with autism spectrum disorder) using these terms: (1) MPH effect on mouse substantia nigra^27^—GSE33619, (2) Psychostimulant (MPH, amphetamine and methamphetamine) effects on rat striatum and prefrontal cortex^28^—GSE33619, and (3) MPH effects on human lymphoblastoids^29^—GSE52889. Following this, each of the data sets were uploaded onto the Pathway Studio software (Elsevier; v. 11.4.08) to run the gene expression profiling and statistics correcting for multiple testing with Benjamin–Hochberg (FDR). The significant (*p* < 0.05) mRNA profiles found to alter as a consequence of MPH treatment were investigated for their enrichment in pathways and functional groups using the Gene-Set Enrichment Analysis (GSEA) tool of the Pathway Studio software (Elsevier; Mammal database), which calculates statistical significance of concordant differential expression for set of genes in pathways, groups, and predefined gene sets between two biological states using ratio (log-ratio) values of genes. Significant results (*p* < 0.0005) for enrichment analysis are presented in Supplementary Material S1. Gene Ontology (GO), pathways, diseases and signal processing related to brain or neuronal processes were marked in green, while Wnt-pathway was marked in blue (Supplementary Table S1).

### Data availability

The data that support the findings of this study are available from the corresponding author upon request.

## Results

### MPH effects on embryonic mNSC differentiation

In order to expand on our previous pilot study^20^, we examined the acute effect of a range of MPH doses up to 100 μM on embryonic mNSC differentiation. MPH treatment at 1 nM and up to 100 nM enhanced cell differentiation into immature neurons detected by positive staining for neuron-specific class III β-tubulin (Tuj1) (Fig. 1; Supplementary Fig. S1), similar to our previous pilot study^20^. We expanded further on this finding and demonstrated that increasing MPH doses up to 100 μM further increased immature neurons density to 207 ± 39% of control (Mann–Whitney test *U* = −2.021, Asymp. *p* = 0.043, Exact *p* = 0.057; Fig. 1). Additionally, mNSC treated with MPH in all doses did not result in a significantly different number of cells differentiating into astrocytes (Glial fibrillary acidic protein/ GFAP-positive staining) compared to control (Fig. 1). On the contrary, a tendency toward an inhibitory effect was observed when treating the cells with the lower dose of 1–100 nM MPH.

### Influence of MPH on rat PC12 cells proliferation and differentiation

Following the possible influence of MPH on cell development, as found in the mNSC, we tested the effects of MPH on the proliferation of PC12 cells (Fig. 2). In order to evaluate cell proliferation in a non-invasive and non-biased manner, the real-time cell impedance automatic monitoring (xCELLigence system) was used for a duration of 72 h to evaluate the first significant time-point at which MPH caused alterations (Fig. 2b and Supplementary Fig. S2). Accordingly, 1–100 nM MPH significantly inhibited PC12 cell proliferation starting 16 h after treatment, which was further confirmed by assessing the 5-bromodeoxyuridine (BrdU) positive cells (Fig. 2c). In order to ensure that the effect did not arise from cell death, apoptosis and cytotoxicity were measured, and demonstrated negligible signals after MPH treatment (Supplementary Fig. S2d–e), suggesting that there was no significant cell death due to MPH.

Daily treatment (5 days) of MPH starting from a 10 nM up to a 100 μM dosage, significantly promoted PC12 cell differentiation, as observed by measuring neurite length normalized to the diameter of the soma (Fig. 2d-f).

### Influence of MPH on human SH-SY5Y cells proliferation and differentiation

After testing proliferation and differentiation alterations following MPH treatment in two non-human cellular models (mouse and rat), we confirmed a decrease in the proliferation of SH-SY5Y cells after MPH treatment, as measured both by the real-time impedance cell analyzer and BrdU staining, starting with the lower 1 nM MPH dose (Fig. 3a, c, d). Furthermore, we confirmed that MPH significantly promoted the differentiation of SH-SY5Y cells (Fig. 4). To exclude the possibility that the effects of MPH on proliferation and differentiation were due to the dopamine transporter inhibition, SH-SY5Y cells were treated with the selective dopamine-transporter inhibitor GBR12909. GBR12909 (1 nM up to 10 µM) treatment did not cause a decrease in proliferation as with MPH (Fig. 3d), but rather a slight non-significant increase was observed. GBR12909 (1 nM up to 10 µM) treatment led to a significant decrease in differentiation (Fig. 4d), which was in contrast to MPH treatment effects. Furthermore, higher doses of GBR12909 (doses of 100 µM) caused cell death in both the proliferation and differentiation experiments, suggesting that increased extracellular dopamine might have caused cytotoxicity.

### Activation of Wnt/β-catenin pathway through MPH treatment

Examining whether the observed effects on proliferation and differentiation after MPH treatment were through the activation of the Wnt/β-catenin pathway was investigated by blocking the Wnt-pathway with Dkk1 30 min before MPH treatment. Indeed, Dkk1 treatment caused a significant increase in the proliferation of SH-SY5Y cells (Fig. 3d), which remained constant after MPH treatment (30 min after blocking the pathway). Controlling for Wnt-activation effects using R-spondin 1 treatment resulted in decreased proliferation as observed with MPH alone (Fig. 3d). Differentiation of SH-SY5Y cells was significantly inhibited by Dkk1 treatment 30 min before MPH treatment (Fig. 4d), whilst the activation of the Wnt-pathway with R-spondin 1 significantly increased differentiation similar to MPH treatment alone (Fig. 4d).

**Fig. 5:**
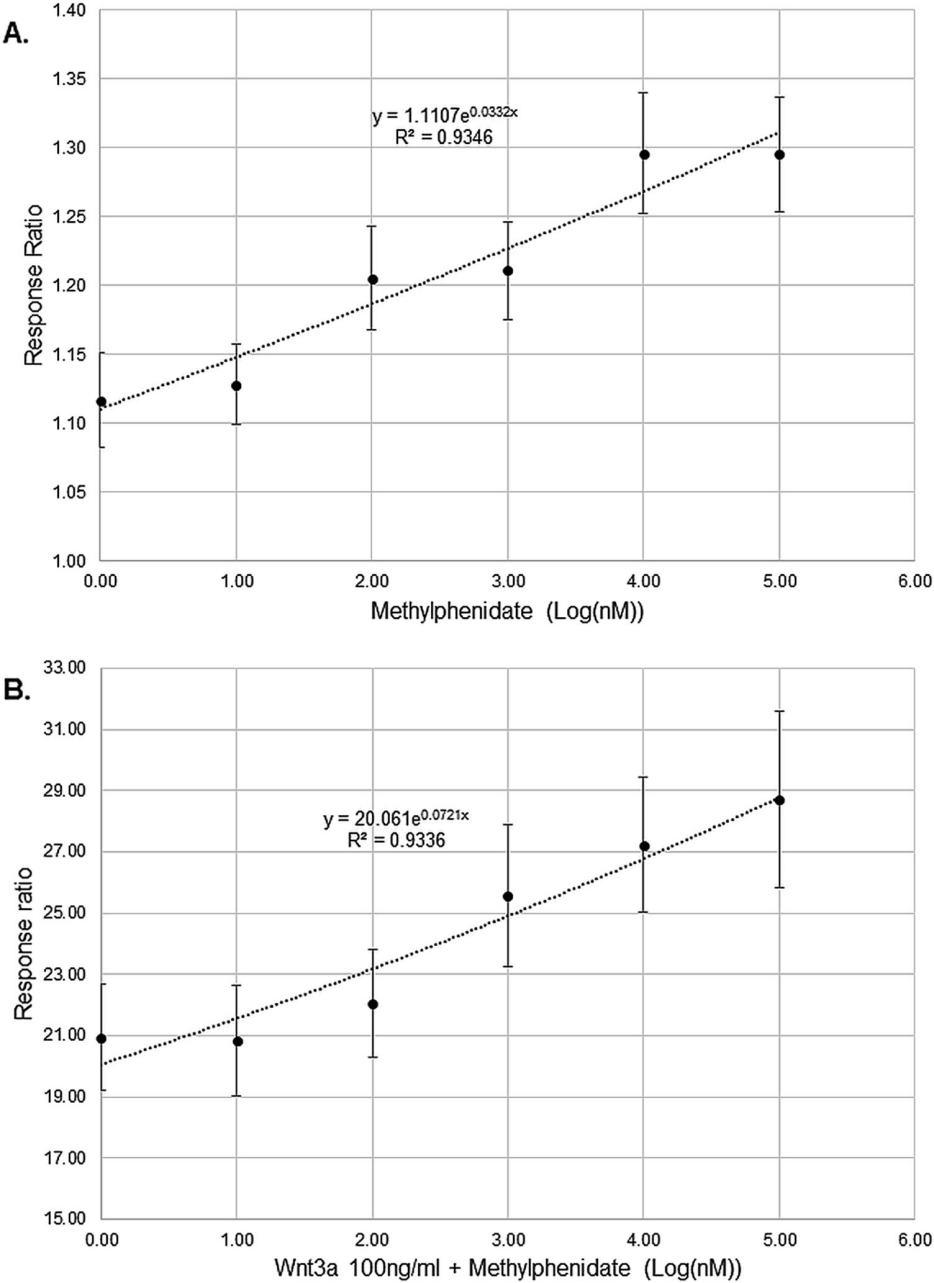
Effect of MPH on the luciferase Wnt reporter assay activation/inhibition. **a** Dose response of MPH on the activation of the leading light Wnt reporter assay represented as response ration (ratio = 1 negative control-water); **b** Dose response of MPH in the presence of Wnt3a (100 ng/ml) on the activation/inhibition of the Leading Light Wnt reporter assay represented as response ration (ratio = 1 negative control-water); *n* = 17–35; Exponential regression was conducted fitting the curve with *R*^2^ > 0.9

In order to verify the activation of Wnt-pathway through MPH treatment, the luciferase Wnt reporter assay was used, showing a dose response activation of the Wnt-pathway through Wnt3a, R-spondin 1 and LiCl (Supplementary Fig. S3a–c), while Dkk1 treatment led to an inhibition of the Wnt-pathway after Wnt3a treatment (Supplementary Fig. S3d). Similarly, MPH from 1 nM up to 100 µM resulted in an exponential increase in Wnt-signaling activation (Fig. 5a). MPH treatment accompanied by 100 ng/ml Wnt3a protein enhanced the already occurring activation (Fig. 5b), in contrast to the inhibitory effect observed for Dkk1 when accompanied with 100 ng/ml Wnt3a (Supplementary Fig. S3d).

LRP, in particularly 5 and 6, is known to activate the canonical Wnt-signaling via binding with the Frizzle (Fz) and Wnt protein^25^. Several mechanisms are existing for regulating Wnt-signaling activation as the inhibitor Dkk1. Furthermore, sclerostin inhibits activation of Wnt-signaling via binding to LRP5/6, which also regulates bone formation^26^. MPH inhibited LRP5 binding to sclerostin (ca, 17%) measured using the Leading Light Sclerostin-LRP interaction screening kit (Enzo Life Sciences Inc. Lausen, Switzerland) in all doses tested (1 nM up to 1 mM) with the strongest inhibition at 1 mM MPH (Supplementary Fig. S4).

### Enrichment analysis for GEO profiles following MPH treatment

We re-analyzed three available gene expression profile data sets extracted from the GEO database, from experiments studying MPH treatment effects in mouse substantia nigra^27^, in rat striatum and frontal cortex^28^, and in immortalized human lymphocytes of controls and adult ADHD patients^29^, in which in the last study cell lines were acutely treated with ca. 111 μM MPH. Pathway enrichment analysis for all significantly altered transcripts was carried out using the GSEA. Indeed, we found some indication that in vivo MPH treatment in mice and rats induced mRNA alterations enriched in pathways involved in cell differentiation and Wnt-signaling, as well as in synaptic, axonal and neuronal processes (Supplementary material S1). The top ranked pathways were of general processes, such as plasma membrane, atlas signaling, cell differentiation, signal transduction etc. followed by the aforementioned pathways with *p*-values lower than 0.0005. In the human lymphoblastoids, enrichment in synaptic, axonal and neuronal processes was observed when comparing between adult ADHD and control cells treated with saline, and in negative regulation of cell proliferation and axonal guidance in cells treated with ca. 111 μM MPH (Supplementary material S1).

## Discussion

This study found that MPH influences proliferation and differentiation processes through Wnt-signaling activation. Evidence for Wnt-signaling activation through MPH treatment was demonstrated using the cell culture model, the luciferase Wnt-reporter assay as well as from GSEA results of three gene expression databases in animals and humans following MPH treatment. In all the above experiments, we repeatedly confirmed that MPH treatment led to an alteration in Wnt-signaling pathways, which at the cellular level promoted cell differentiation concomitantly to reduced proliferation.

Modulation of Wnt-signaling components is known to be induced by cocaine, an addictive psychostimulant. Cocaine has been shown to cause a widespread downregulation of Wnt-signaling molecules in the nucleus accumbens of mice, a key brain region in reward^30^, as well as a downregulation of the Wnt-canonical pathway after induction of cocaine sensitization in the prefrontal cortex of rats, amygdala and dorsal striatum^31^. On the other hand, amphetamine, (another first–line treatment of ADHD, e.g., according to the often used Canadian guidelines (CADDRA) and second-line treatment according to the most other guidelines), reportedly stimulated Wnt3 with an increase in total β-catenin in rat nucleus accumbens^32^. In adult mice with inducible expression of Dkk1, there was a degeneration of cortico-striatal synapses, but amphetamine did not increase locomotion as compared to those treated with saline^33^. However, induced Dkk1 adult mice were already slightly hyperactive compared with control animals^33^, which suggest that developmental timing of such induced imbalance is important in Wnt activity. Although one might hypothesize that psychostimulants effects on Wnt-signaling may be due to monoamine levels or dopamine receptor activity modulation, we did not observe such changes after treatment with the selective dopamine-transporter inhibitor GBR12909. Moreover, an increased dose of GBR12909 led to cell death that was not observed with MPH, even after higher doses. Although this suggests that MPH has a direct interaction with Wnt-pathways, this requires further investigation.

Alterations during neurogenesis and the plasticity of the developing brain, as well as later in life, are often discussed to be vulnerability factors for the development of psychiatric disorders, such as ADHD. Wnt-signaling has been shown to regulate both embryonal neurogenesis, in particular in the dopaminergic system^22^, as well as adult neurogenesis processes mostly known to occur in the hippocampus^23^. Various animal models and paradigms have demonstrated MPHs capability to modulate neurogenesis and brain plasticity^15–17,34^. Recently, personalized fingerprinting of brain functional connectome demonstrated sensitive time points in brain maturation and plasticity, that were distinctively different to those with symptoms such as attention deficit^35,36^. Psychostimulant treatment in ADHD was reported to induce changes in functional brain connectivity, which were also associated with symptom improvements^37,38^. Such alterations need to be further understood in terms of the optimum time point and duration of treatment with psychostimulants to reach best results and benefits.

There is some evidence for candidate risk genes in ADHD to be enriched in pathways such as neurite outgrowth, axon guidance, cell trafficking processes, brain development, neurogenesis, and Wnt/β-catenin pathway^39–41^. Wnt-signaling is known to play an important role in the development and survival of neuronal cells in the CNS. Moreover, growing evidence indicates that Wnt-signaling pathways also regulate the structure and function of the adult nervous system. Wnt-signaling was shown to regulate the formation and function of neuronal circuits by controlling neuronal differentiation, axon outgrowth and guidance, dendrite development, synaptic function, and neuronal plasticity^42,43^. Genome-wide association studies (GWAS) have reported the *KCNIP4* gene, known to be associated with ADHD, plays a role in a negative-feedback loop in the Wnt/β-catenin pathway^44^. Several GWAS and candidate gene studies describe the association of *CDH13* (encodes the cadherin-13 protein) and *CTNNA2* (encodes the α2-catenin protein) genes with ADHD or with associated phenotypes (e.g., hyperactivity/impulsivity symptoms; verbal working-memory)^45–47^ pointing to alterations in the Wnt-associated genes in ADHD. Furthermore, an enrichment analysis of a GWAS analyzing the association in a subpopulation of oppositional defiant disorder in ADHD found the β-catenin to be significantly highlighted^48^. In the often co-morbid disorder, autism spectrum disorder (ASD), evidence from human genetic data to animal genetic models also point to the involvement of Wnt-pathway (see review in ref. 49). In summary, this evidence of dysregulation of Wnt-pathways in ADHD might also predict the efficacy of MPH treatment in some patients compared to non-responders. However, this needs further investigation using patient specific cellular models, i.e., induced pluripotent stem cells (iPSC).

In conclusion, this study has shed some light on to the additional targets of MPH and its influence on cell proliferation and differentiation. Wnt-signaling alterations through MPH points to the importance of gaining further understanding of its short-term and long-term effects and duration of treatment. Studies using patients’ specific neuronal models will greatly improve the accessibility of human tissue, which is currently limited due to scarce postmortem samples from ADHD patients.

## Electronic supplementary material

Supplementary files
