# Supplementary material for: Methylphenidate enhances neuronal differentiation and reduces proliferation concomitant to activation of Wnt signal transduction pathways
Source: Transl Psychiatry. 2018 Mar 1;8:51. doi: 10.1038/s41398-018-0096-8 (PMC5830437; doi:10.1038/s41398-018-0096-8)
Supplement: Supplementary file 1 — Supplementary files [file 41398_2018_96_MOESM1_ESM.pdf]

Supplementary material

Methylphenidate enhances neuronal differentiation and reduces proliferation concomitant to activation of Wnt signal transduction pathways

Edna Grünblatt[1-3*✉], Jasmin Bartl[1,4*], Susanne Walitza[1-3]

Supplementary Material and Methods

1. Preparation of compound solution

Cells were treated with varying concentrations (1 nM – 100 µM) of d/l-threo methylphenidate (MPH) (cat. no. M2892; Sigma Aldrich, Switzerland) or the vehicle (water) as control. MPH was protected from light during all treatments and the dilutions were always prepared freshly prior to use to avoid any auto-degradation. GBR12909 (cat. no. D052; Sigma Aldrich, Switzerland) was diluted in DMSO and cells were treated in varying concentrations (1 nM – 10 µM) or with DMSO as a control. R-spondin 1 (100 ng/ml) and dickkopf-protein 1 (200 ng/ml) (dkk-1; cat. no. cat. no. SRP6487 & SRP3258; Sigma Aldrich, Switzerland) were diluted in water.

2. Murine neuronal stem cell (mNSC) sphere monolayer culturing and differentiation study

2.1. Immunocytochemistry

Fixed and prepared cells were stained for differentiation studies with rabbit monoclonal antibody against glial fibrillary acidic protein (GFAP; 1:200, Sigma aldrich, USA, Cat-No: C4546) and/or with mouse monoclonal antibody against β-tubulin III (Tuj 1; 1:300, Sigma aldrich, USA, Cat-No: T3952) overnight at 4°C. For total cell count, nuclei were visualized using Hoechst 33258 staining (1:100; Invitrogen, Cat-No: H3569) for 15 minutes at room temperature (RT). After overnight incubation, the primary antibodies were visualized with secondary antibodies against rabbit or mouse conjugated to the following fluorochromes: Alexa FluorW -488 and Alexa FluorW -555 (ThermoSchientific, Switzerland). Staining was visualized under the inverted microscope (Olympus IX81, Germany) with DP72 Digital camera and the xcellence software (Olympus, Germany). In Supplementary Fig. S1 examples for staining and overlays are shown.

For each well, first the total number of cells within the well was determined manually counting Hoechst positive stained cells. Secondly, GFAP positive or Tuj 1 positive cells were manually counted, respectively. Subsequently, the relative ratio of positive cells (sum of GFAP or Tuj 1 positive cells, respectively) to the total cell count (sum of Hoechst-stained cells) was calculated. In order to present results in comparison to the untreated cells, percentages were calculated for each treatment dose, setting the control as 100%.

3. Neuronal cell line culture

3.1. Culturing of proliferating SH-SY5Y and PC12 cells

Human neuroblastoma SH-SY5Y cells (European Collection of Authenticated Cell Cultures (ECACC), UK) were grown in buffered Dulbecco`s modified Eagle medium (DMEM/F-12) (Sigma Aldrich, Switzerland) supplemented with 10% fetal bovine serum (FBS; Thermo Fisher Scientific, Switzerland) in a humidified incubator (5% CO_2) at 37°C. Rat pheochromocytoma (PC12) cells (ECACC, UK) were grown in buffered 10 mg/ml glucose Dulbecco's modified Eagle medium (DMEM) (Thermo Fisher Scientific, Switzerland) supplemented with 10% FBS (Thermo Fisher Scientific, Switzerland), 5% horse serum (Thermo Fisher Scientific, Switzerland), and 0.3% gentamycin (50 mg/ml) (Thermo Fisher Scientific, Switzerland) in a humidified incubator (5% CO_2) at 37°C.

3.2. Differentiation of SH-SY5Y and PC12 cells

SH-SY5Y cells were seeded in poly-D-lysine/laminin coated 8-well slides (Corning, Switzerland) at a density of 10^4cells/well. The cells were grown at 37°C, 5% CO_2 for 24 hours in normal growth medium before it was exchanged with a differentiation medium (DM), consisting of a normal growth medium supplemented with 10 µM retinoic acid (Sigma Aldrich, Switzerland) and 50 ng/ml nerve growth factor (Sigma Aldrich, Switzerland). PC12 cells were seeded in Collagen I (ScienCell, USA) coated 4-well slides (Corning, Switzerland) at a density of 10^5cells/well. The cells were grown at 37°C, 5% CO_2 for 24 hours in normal growth medium before it was exchanged with DM, consisting of a 1:5 mixture of normal growth medium and DMEM supplemented with 100 ng/ml nerve growth factor. DM was exchanged every second day.

3.3. BrdU Staining

Cells were fixed with 4% ice-cold paraform aldehyde (SantaCruz Biotechnologies, Switzerland) for 20 minutes at RT. This was followed by incubation in 1 M HCl on ice for 10 minutes, followed by 2 M HCl at RT for 20 minutes and another 20 minutes in 2 M HCl at 37°C, in order to enable breaking of the nucleus and denaturing the DNA. After neutralizing with 1.5 M sodium borate buffer for 10 minutes, the cells were washed trice with PBS. To prevent unspecific binding, the cells were blocked in a blocking buffer (PBS, 1% goat serum and 0.03 % triton-X- 100) for 90 minutes. Alexa Fluor 488®-linked anti-BrdU antibody (Millipore, Switzerland) was added to the cells, which were then incubated in the dark overnight at 4°C. The cells were washed with PBS three times for five minutes. For the mounting a DAPI (nucleus staining reagent)-containing mounting medium (Abcam, UK) was used. The slide was dried over night before pictures were taken at 20x magnification under the inverted microscope (Olympus IX81, Germany) with DP72 Digital camera. Cells were counted using the xcellence software (Olympus, Germany).

4. Activity detection of canonical Wnt-signaling with luciferase Wnt reporter assay

To confirm our pharmacological analysis of the Wnt-signaling activation by MPH and R-Spondin1 against Wnt-signaling inhibition via dkk1, we used the Leading Light® Wnt Reporter Assay (Enzo Life Sciences, Lausen, Switzerland). This assay, is a cell-based luciferase activity test conducted in a 96-well plate, relaying on an engineered 3T3 mouse fibroblast cell line, which expresses the firefly luciferase reported gene under the control of Wnt-responsive promoters (TCF/LEF). Dose-dependent up-regulation of luciferase activity can be achieved by adding Wnt protein or Wnt agonist, while down-regulation is achieved by addition of Wnt antagonist. Wnt reporter cell lines were grown in 4500mg/L glucose Dulbecco‘s modified Eagle medium (DMEM) (Sigma, Switzerland) supplemented with 10% FBS (Thermo Fisher Scientific, Switzerland), 4mM L-Glutamine (Life Technologies) and 1mM Sodium Pyruvate (Life Technologies, Switzerland). 50µl cells (15,000 cells/well) in growth medium were added to the 96-well plate. Plates were incubated overnight in humidified 37ºC and 5% CO_2 incubator.

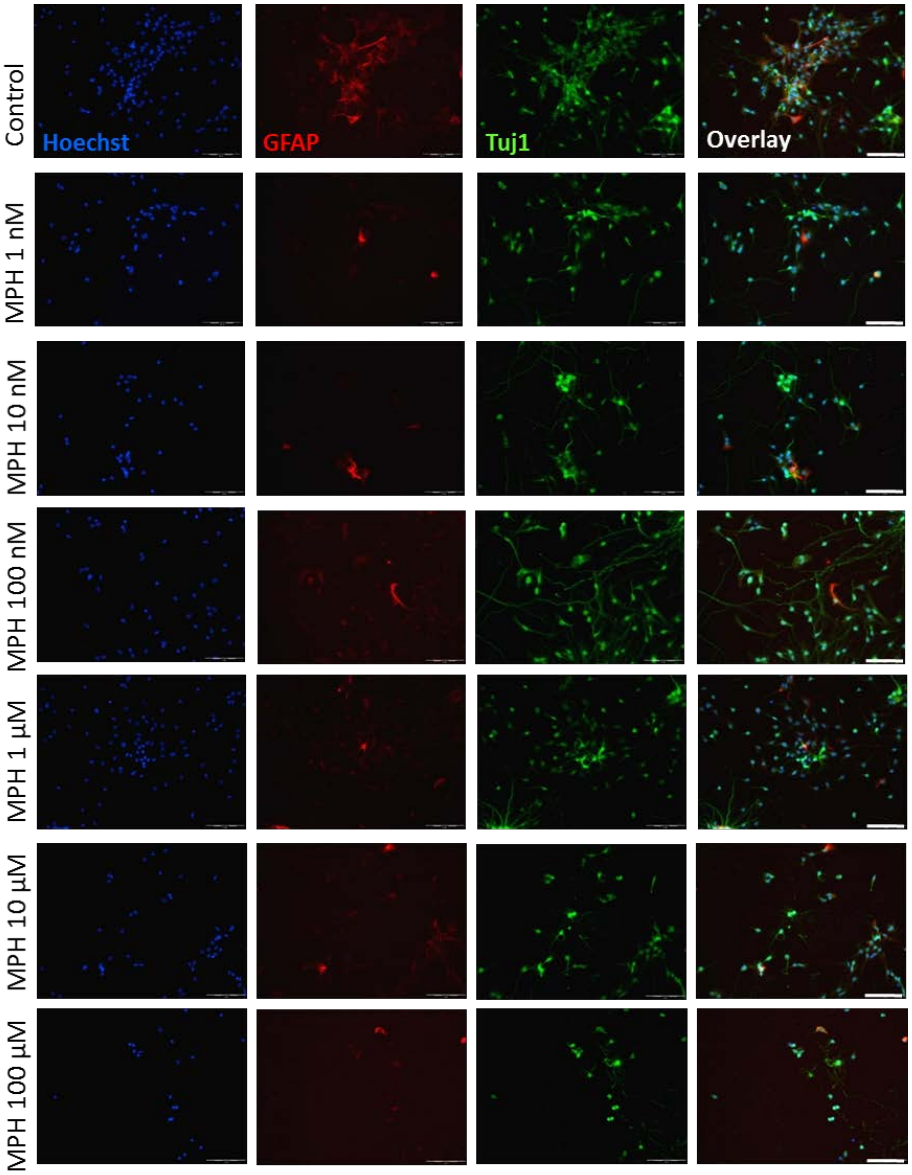

Supplementary Figure S1: Effect of methylphenidate (MPH) on embryonic murine neuronal stem cell (mNSC) differentiation into astrocyte (GFAP$^+$ cells) and neurons (Tuj1$^+$ cells). Embryonic mNSC were treated with MPH (1 nM up to 100 µM) or with vehicle (control). The cell number was determined via nucleus staining using Hoechst four days after treatment with MPH, while differentiation into astrocytes was determined by positive staining to the glial fibrillary acidic protein (GFAP) and differentiation into immature neurons was determined by positive staining to the neuron-specific class III beta-tubulin (Tuj1) compared to the total number of cells. Representative immunocytochemistry staining's for each of the treatments and control of embryonic mNSC against GFAP (red), Tuj 1 (green) and for total cell count using Hoechst 33258 staining (blue). Scale bar = 50 µm.

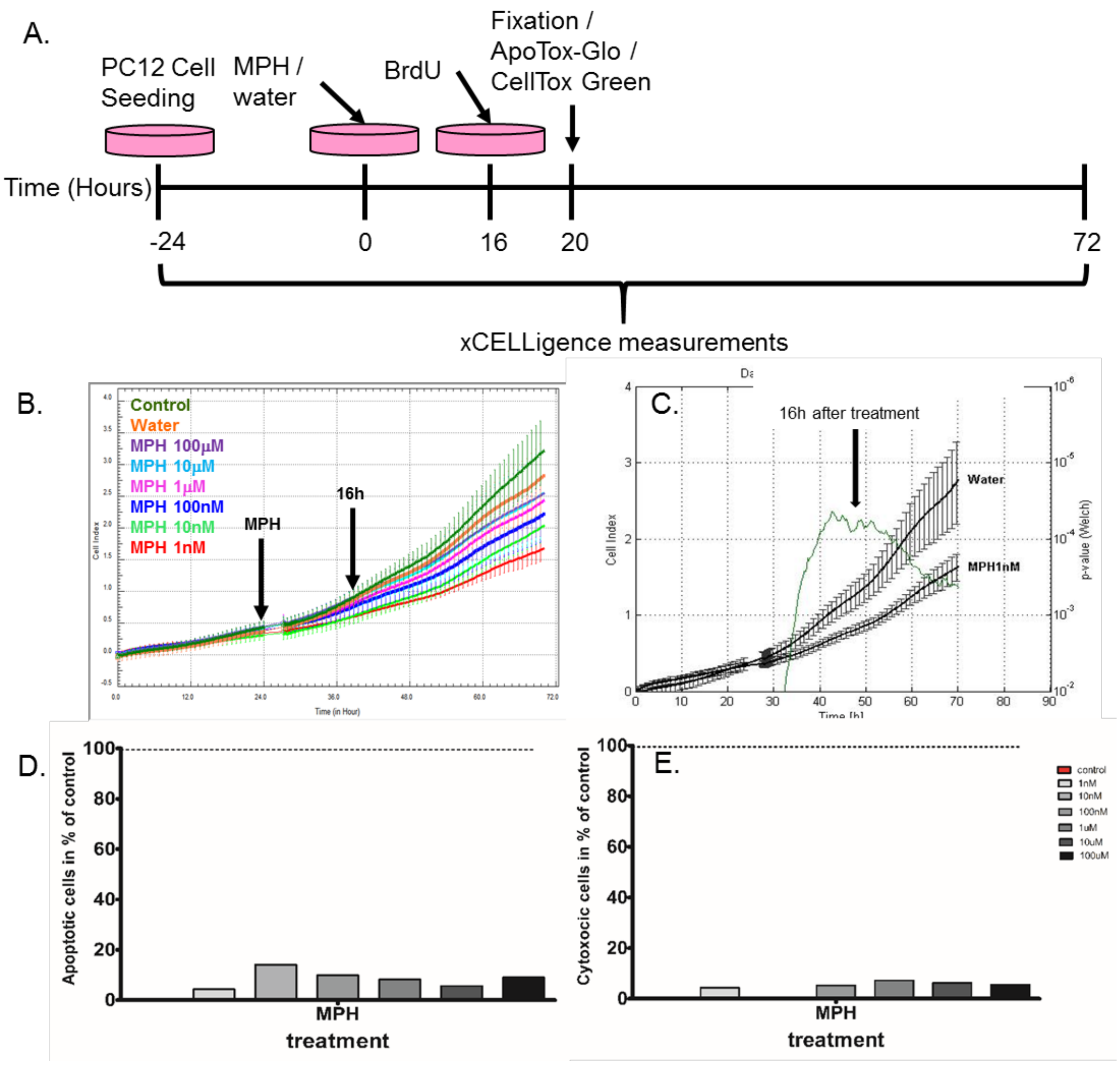

Supplementary Figure S2: Influence of methylphenidate (MPH) treatment on PC12 cells proliferation and possible toxicity. A. The time line of cell culturing, treatment and measurements. B. Representative xCELLigenece run measuring in real-time impedance of cell proliferation (given as mean cell index on y-axis) before and after MPH (1nm-100µM) or vehicle (water) treatment during 72 h (x-axis). C. A representative statistical analysis conducted in MATLAB showing vehicle vs. MPH 1nM as in (B) with an overlay (green line) of the p-value according to Welch-test (right y-axis). (D) Mean apoptosis (ApoTox-Glo) measured as % of control ± SEM at 20 h after MPH treatment showing negligible apoptosis signal. (E) Mean cytotoxicity (CellTox Green) measured as % of control ± SEM at 20 h after MPH treatment showing negligible cytotoxicity signal.

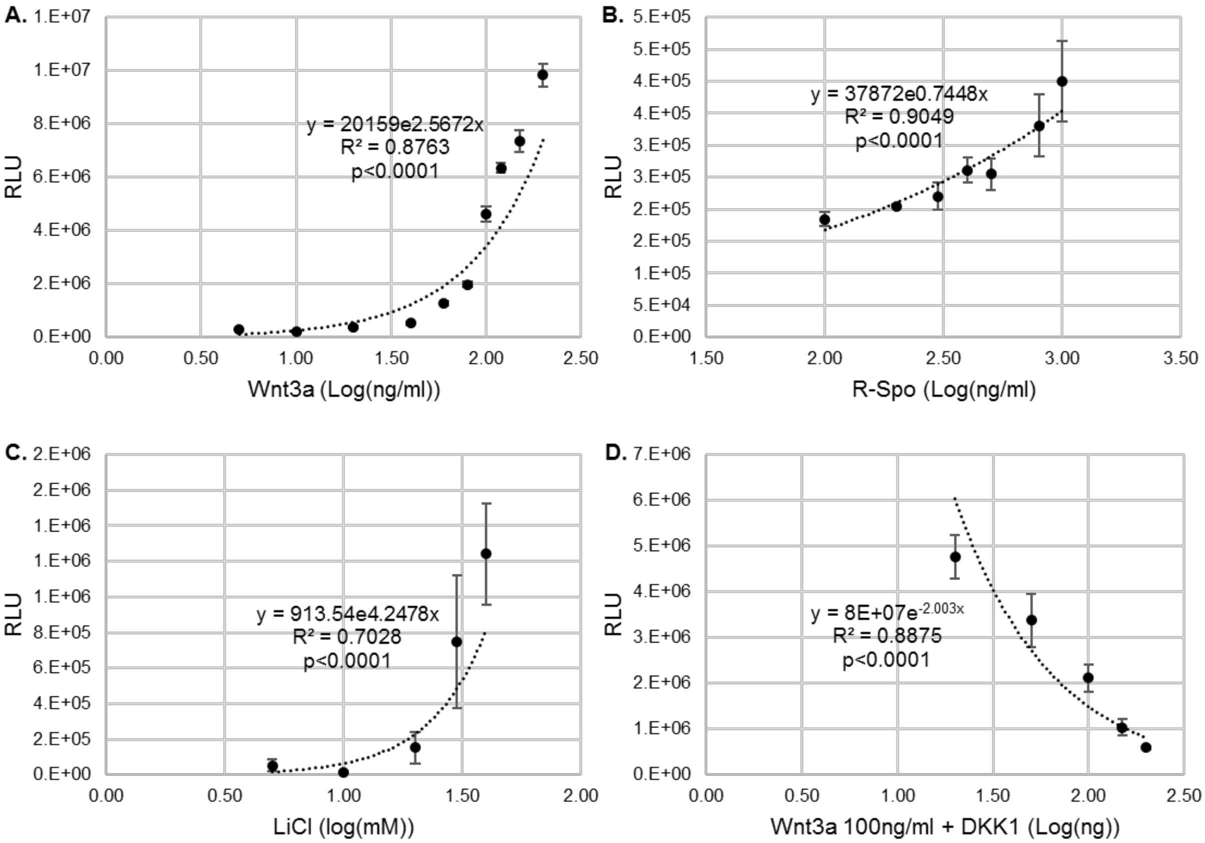

Supplementary Figure S3: Effect of Wnt activators (Wnt3a, R-Spondin 1, and LiCl) and an inhibitor (Dkk1) on luciferase Wnt reporter assay activation / inhibition. (A) Dose response of Wnt3a protein on the activation of the Leading Light Wnt reporter cells. n=4-22; (B) Dose response of R-spondin 1 (R-Spo) on the activation of the Leading Light Wnt reporter cells. n=3-7; (C) Dose response of LiCl on the activation of the Leading Light Wnt reporter cells. n=3-19; (D) Dose response of Dkk1 protein in the presence of Wnt3a (100 ng/ml) on the inhibition of the Leading Light Wnt reporter cells. n=9-22. Exponential regression was conducted fitting the regression curve with R^2>0.7.

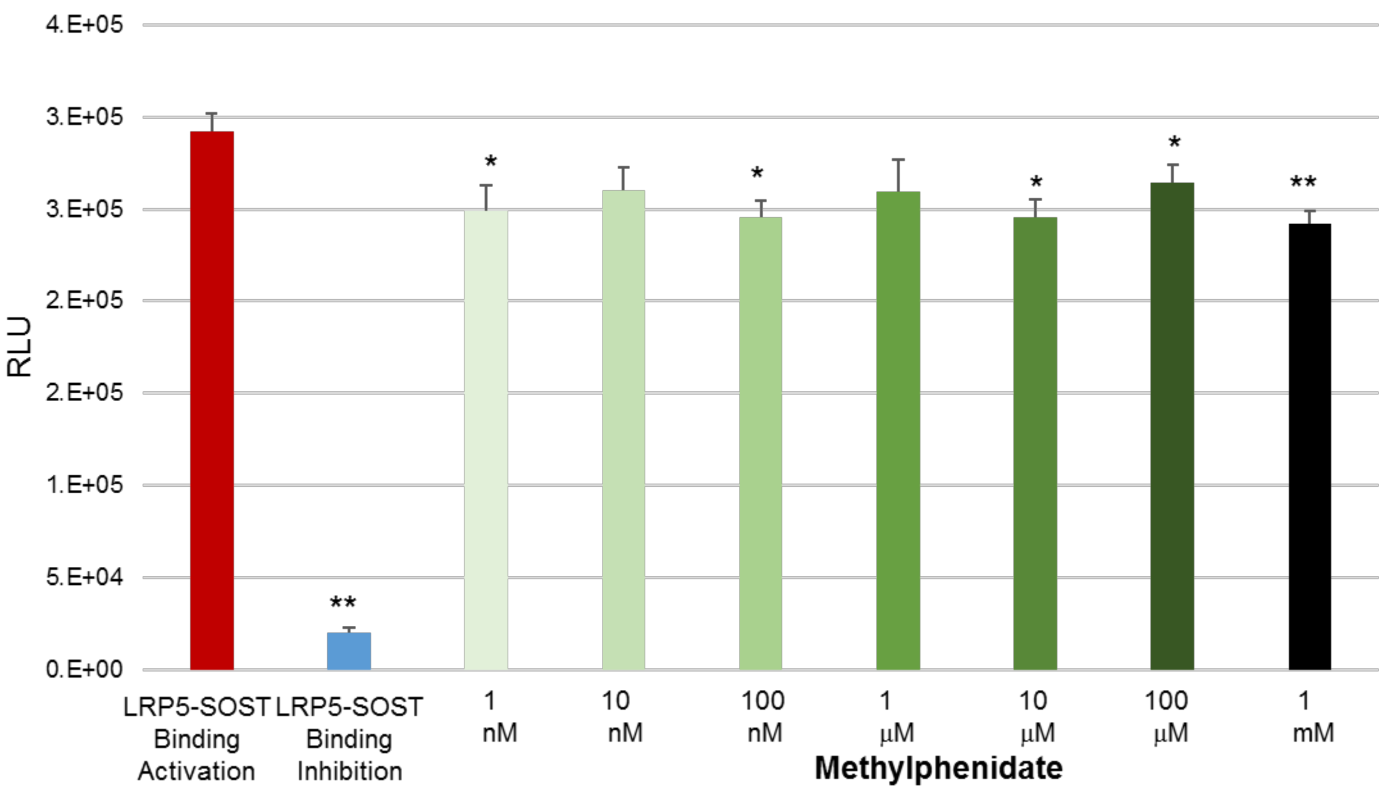

Supplementary Figure S4: Effect of methylphenidate on the interaction of sclerostin (SOST) with LRP5 tested by the Leading Light® Sclerostin-LRP interaction screening kit (Enzo Life Sciences Inc. Lausen, Switzerland). Positive binding between sclerostin and LRP5 following addition of LRP5-Alkaline phosphatase protein induced high luminescence signal (red bar), while addition of the Acid Green 25 (binding inhibitor) resulted in signal reduction (ca. 6% of the binding activation is left) (blue bar). Addition of methylphenidate starting from 1nM up to 1 mM reduced the signal to maximum of 17% (green bars). Bars represent mean RLU ± SEM (n=5-6). Kruskal-Wallis (F=8, H=22.380 p=0.0043), followed by Mann-Whitney-test * $p<0.05$ ** $p<0.01$ versus binding activation.

Supplementary Table S1: Gene Set Enrichment Analysis (GSEA; using the software Pathway Studio® version 114.0.8) of the available database for gene expression studies involving methylphenidate treatment in mammals.

1) Sadasivan S et al. 2012 [1]: Swiss Webster mice (3 weeks old), i.p. treated with saline, 1 mg/kg or 10 mg/kg methylphenidate once daily- for 12 weeks, 7 days' washout. Substantia nigra mRNA using Affymetrix HT MG-430 PM array.
Database on GEO: GSE33691

Chronic 1mg/kg MPH Mouse SN

Name	# of Entities	Expanded # of Entities	# of Measured Entities	Median change	p-value	Hit type
multicellular organismal development	1171	1171	1073	-1.001770694	2.25296E-12	biological_process
plasma membrane	5390	5390	3575	-1.005534712	2.09322E-11	cellular_component
integral component of plasma membrane	1360	1360	1101	-1.005092317	1.00697E-10	cellular_component
proteinaceous extracellular matrix	349	349	310	1.003269325	3.13082E-10	cellular_component
Atlas of Signaling	380	2028	1245	-1.002716707	9.4145E-10	Cell Signaling
cell differentiation	849	849	755	-1.000402833	1.39388E-06	biological_process
neuropeptide signaling pathway	106	106	90	-1.019128624	3.70092E-06	biological_process
cell surface	645	645	542	-1.004494091	4.5826E-06	cellular_component
axon guidance	385	385	371	-1.005587771	5.87638E-06	biological_process
dendrite membrane	22	22	22	1.023333506	1.35323E-05	cellular_component
neural crest cell development	15	15	13	1.058296741	4.13872E-05	biological_process
neuron fate specification	14	14	13	-1.034779373	5.1894E-05	biological_process
synaptic transmission	472	472	428	-1.004584643	7.84504E-05	biological_process
social behavior	52	52	46	1.020725612	0.000101242	biological_process
Wnt signaling pathway	231	231	193	1.006366167	0.000227649	biological_process
synapse assembly	55	55	48	-1.005885398	0.000328795	biological_process

Chronic 10mg/kg MPH Mouse SN

Name	# of Entities	Expanded # of Entities	# of Measured Entities	Median change	p-value	Hit type
Atlas of Signaling	380	2028	1245	-1.000721214	6.33031E-14	Cell Signaling
extracellular region	2250	2250	1651	-1.002486493	1.40597E-11	cellular_component
proteinaceous extracellular matrix	349	349	310	1.010965241	1.04278E-10	cellular_component
plasma membrane	5390	5390	3575	-1.004804104	2.81937E-10	cellular_component
integral component of plasma membrane	1360	1360	1101	-1.004003321	4.38301E-09	cellular_component
cell differentiation	849	849	755	-1.004708505	1.65844E-05	biological_process
neuropeptide signaling pathway	106	106	90	1.00459659	1.73489E-05	biological_process
neuromuscular synaptic transmission	19	19	18	1.039138796	6.63467E-05	biological_process
synaptic transmission	472	472	428	-1.003749126	0.000188697	biological_process

2) Dela Pena I et al. 2013 [2]: Male Wistar rats (6 weeks old), i.p. treated 2x daily for 7 days: with saline (C1), 5 mg/kg methylphenidate (C1.1). Following CPP- group with self-administration for 8-days or either Saline or MPH (C2). Striatum and Prefrontal cortex (PFC) mRNA using Affymetrix Rat Genome 230 2.0 array.
Database on GEO: GSE43748

5mg/kg MPH rat striatum C1.1 vs saline C1

Name	# of Entities	Expanded # of Entities	# of Measured Entities	Median change	p-value	Hit type
extracellular region	2250	2250	1331	-1.045058812	8.05471E-32	cellular_component
extracellular space	1557	1557	1019	-1.048719144	1.59526E-19	cellular_component
integral component of plasma membrane	1360	1360	963	-1.080850862	6.27611E-16	cellular_component
sequence-specific DNA binding	645	645	453	-1.115796201	9.14035E-15	molecular_function
plasma membrane	5390	5390	3130	-1.078799144	3.38168E-14	cellular_component
Biofluids assayable substances	4140	9263	3392	-1.064087529	8.5269E-14	Pathway Studio Ontology
signal transduction	1843	1843	1365	-1.075349382	2.2553E-13	biological_process
TGFB1-ACVRL1 Expression Targets	221	230	208	-1.038256586	9.21814E-13	Expression Targets Pathways
G-protein coupled receptor signaling pathway	2567	2567	463	-1.098828607	4.37575E-12	biological_process
G-protein coupled receptor activity	1768	1768	301	-1.140718965	1.17771E-11	molecular_function

hormone activity	152	152	105	-1.053268852	2.85489E-11	molecular_function
transcription factor activity, sequence-specific DNA binding	1125	1125	731	-1.07943767	3.6269E-11	molecular_function
transcriptional activator activity, RNA polymerase II core promoter proximal region sequence-specific binding	267	267	211	-1.126835414	5.15856E-11	molecular_function
external side of plasma membrane	288	288	210	-1.073643514	8.56879E-11	cellular_component
Proteins Involved in Pathogenesis of Atherosclerosis	201	212	165	1.014999983	9.50404E-11	Disease Collections
signal transducer activity	739	739	474	-1.102954853	1.0978E-10	molecular_function
Insulin/CEBPA/CTNNB/FOXA/FOXO Expression Targets	145	157	138	-1.054490618	1.73111E-10	Expression Targets Pathways
immune response	468	468	264	1.039052844	1.89774E-10	biological_process
Proteins Involved in Pathogenesis of Arterial Hypertension	257	281	234	-1.06174063	5.37103E-10	Disease Collections
multicellular organismal development	1171	1171	843	-1.0678359	6.00508E-09	biological_process
cell differentiation	849	849	628	-1.071770248	1.57668E-07	biological_process

5mg/kg MPH rat striatum C2 vs saline C2

Name	# of Entities	Expanded # of Entities	# of Measured Entities	Median change	p-value	Hit type
extracellular region	2250	2250	1331	1.123785391	5.19614E-37	cellular_component
extracellular space	1557	1557	1019	1.105057259	2.52616E-25	cellular_component
plasma membrane	5390	5390	3130	1.059837403	9.81867E-24	cellular_component
integral component of plasma membrane	1360	1360	963	1.042840131	3.43473E-20	cellular_component
G-protein coupled receptor signaling pathway	2567	2567	463	1.101812067	2.22145E-17	biological_process
signal transduction	1843	1843	1365	1.095857912	1.72332E-16	biological_process
G-protein coupled receptor activity	1768	1768	301	1.095857912	1.03716E-15	molecular_function
Atlas of Signaling	380	2028	1039	1.085267142	1.57994E-15	Cell Signaling
Biofluids assayable substances	4140	9263	3392	1.074778808	6.96678E-15	Pathway Studio Ontology
sequence-specific DNA binding	645	645	453	1.125952007	9.13462E-15	molecular_function
multicellular organismal development	1171	1171	843	1.11746541	1.049E-14	biological_process

Secreted proteins	7555	12985	6654	1.056185806	2.05585E-14	Pathway Studio Ontology
external side of plasma membrane	288	288	210	1.10983508	3.43E-13	cellular_component
Proteins Involved in Pathogenesis of Arterial Hypertension	257	281	234	1.116239899	1.0469E-11	Disease Collections
IGF1/MEF/MYOD/MYOG Expression Targets	135	148	132	1.151018415	1.24432E-11	Expression Targets Pathways
signal transducer activity	739	739	474	1.093796224	2.39635E-11	molecular_function
transcriptional activator activity, RNA polymerase II core promoter proximal region sequence-specific binding	267	267	211	1.161696575	3.70286E-11	molecular_function
TGFB1-TGFBR1/AP-1 Expression Targets	123	134	122	1.174460534	5.54533E-11	Expression Targets Pathways
TGFB1-ACVRL1 Expression Targets	221	230	208	1.150224108	6.75501E-11	Expression Targets Pathways
TGFB1-TGFBR2 Expression Targets	116	122	108	1.159089702	1.01171E-10	Expression Targets Pathways
Proteins Involved in Pathogenesis of Atherosclerosis	201	212	165	1.216120358	1.52405E-10	Disease Collections
synaptic transmission	472	472	418	1.03481214	2.47204E-10	biological_process
IGF1/STAT Expression Targets	107	113	106	1.154541884	2.5247E-10	Expression Targets Pathways
proteinaceous extracellular matrix	349	349	263	1.175787989	2.69761E-10	cellular_component
cell-cell signaling	269	269	218	1.151018415	3.25009E-10	biological_process
IL1B Expression Targets	169	188	162	1.13998323	3.66446E-10	Expression Targets Pathways
EGF/CTNN Expression Targets	143	152	139	1.151018415	4.64596E-10	Expression Targets Pathways
ion transmembrane transport	291	291	239	1.098950927	9.37338E-10	biological_process
immune response	468	468	264	1.113115538	9.64808E-10	biological_process
cell differentiation	849	849	628	1.101692694	7.9454E-09	biological_process
postsynaptic membrane	227	227	203	1.096269846	1.25526E-06	cellular_component

5mg/kg MPH rat PFC C1.1 vs saline C1

Name	# of Entities	Expanded # of Entities	# of Measured Entities	Median change	p-value	Hit type
extracellular region	2250	2250	1331	-1.046290799	4.94976E-26	cellular_component
extracellular space	1557	1557	1019	-1.051940289	1.57008E-15	cellular_component
G-protein coupled receptor activity	1768	1768	301	-1.101329228	1.47712E-14	molecular_function

integral component of plasma membrane	1360	1360	963	-1.089762753	3.89799E-14	cellular_component
plasma membrane	5390	5390	3130	-1.081730797	5.81395E-14	cellular_component
immune response	468	468	264	-1.001758461	1.13252E-13	biological_process
G-protein coupled receptor signaling pathway	2567	2567	463	-1.074775622	1.17876E-11	biological_process
signal transduction	1843	1843	1365	-1.07424468	1.37001E-11	biological_process
positive regulation of transcription from RNA polymerase II promoter	1041	1041	842	-1.086144211	5.77276E-11	biological_process
Biofluids assayable substances	4140	9263	3392	-1.034780535	5.99451E-11	Pathway Studio Ontology
sequence-specific DNA binding	645	645	453	-1.067247492	1.57115E-10	molecular_function
multicellular organismal development	1171	1171	843	-1.067963324	2.49388E-08	biological_process
synaptic transmission	472	472	418	-1.130977952	2.23372E-07	biological_process
cell differentiation	849	849	628	-1.07141252	1.12163E-06	biological_process
Canonical WNT Signaling Expression Targets	47	69	55	-1.101923998	2.31766E-05	Expression Targets Pathways
regulation of neuron differentiation	39	39	33	-1.296546646	8.83452E-05	biological_process

5mg/kg MPH rat PFC C2 vs saline C2

Name	# of Entities	Expanded # of Entities	# of Measured Entities	Median change	p-value	Hit type
extracellular region	2250	2250	1331	1.073552014	1.05216E-35	cellular_component
extracellular space	1557	1557	1019	1.089045295	2.37554E-31	cellular_component
integral component of plasma membrane	1360	1360	963	1.07933207	1.42277E-22	cellular_component
G-protein coupled receptor activity	1768	1768	301	1.021735941	1.85815E-18	molecular_function
G-protein coupled receptor signaling pathway	2567	2567	463	1.054024962	5.50181E-18	biological_process
plasma membrane	5390	5390	3130	1.071083099	6.19118E-18	cellular_component
signal transduction	1843	1843	1365	1.083165255	1.83353E-16	biological_process
Biofluids assayable substances	4140	9263	3392	1.067314879	1.51282E-14	Pathway Studio Ontology
external side of plasma membrane	288	288	210	1.091184159	1.88365E-12	cellular_component
hormone activity	152	152	105	1.177512318	2.47992E-12	molecular_function
signal transducer activity	739	739	474	1.057149949	6.93254E-12	molecular_function

positive regulation of cytosolic calcium ion concentration	147	147	129	1.136846448	7.64233E-12	biological_process
sequence-specific DNA binding	645	645	453	1.12714443	5.72771E-11	molecular_function
Atlas of Signaling	380	2028	1039	1.081892397	1.33707E-10	Cell Signaling
Proteins Involved in Pathogenesis of Arterial Hypertension	257	281	234	1.098441227	2.10116E-10	Disease Collections
negative regulation of endopeptidase activity	223	223	116	1.151284071	3.48051E-10	biological_process
Secreted proteins	7555	12985	6654	1.059808269	3.80377E-10	Pathway Studio Ontology
cell-cell signaling	269	269	218	1.094308674	4.63015E-10	biological_process
cell differentiation	849	849	628	1.085972476	2.19728E-08	biological_process
multicellular organismal development	1171	1171	843	1.059005854	2.78393E-08	biological_process

3) Schwarz R et al. 2015 [3]: 180: Human lymphoblastoid cells (LCL) from adult ADHD (aADHD; 5 males 4 females) and healthy controls (2 males 7 females). 30ng/µl MPH (Mw=269.77 g/mol; 111.2µM) treatment or vehicle for 1h, and chronic after 2 weeks. LCL mRNA pool (all samples together) using Affymetrix Human Genome U133 Plus 2.0 array.
Database on GEO: GSE52889

Human LCL aADHD MPH vs. Saline

Name	# of Entities	Expanded # of Entities	# of Measured Entities	Median change	p-value	Hit type
nucleoplasm	2669	2669	2518	-1.00722603	2.66938E-38	cellular_component
nucleus	6877	6877	5961	-1.002796865	5.69797E-32	cellular_component
cytoplasm	6831	6831	6084	-1.001717133	3.9469E-22	cellular_component
nucleotide binding	1946	1946	1792	-1.004093337	2.59455E-17	molecular_function
regulation of transcription, DNA-templated	2670	2670	2279	1.00486156	2.77711E-16	biological_process
transcription, DNA-templated	2430	2430	2206	1.005069899	1.33166E-15	biological_process
cytosol	3173	3173	3034	-1.002656374	1.10898E-14	cellular_component
metal ion binding	3566	3568	3178	1.00150887	9.57377E-13	molecular_function
Golgi apparatus	1245	1245	1151	-1.002474234	4.82146E-12	cellular_component
poly(A) RNA binding	1254	1254	1109	-1.009325635	6.12536E-12	molecular_function
DNA binding	2411	2411	2103	1.002913502	7.75104E-11	molecular_function

nucleic acid binding	1094	1094	856	-1.001676871	1.06298E-10	molecular_function
Proteins Involved in Pathogenesis of Melanoma	245	264	240	1.007240057	4.77857E-10	Disease Collections
ATP binding	1601	1601	1422	-1.002953279	6.15091E-10	molecular_function
zinc ion binding	1359	1359	1173	1.003697079	8.0144E-10	molecular_function
Proteins Involved in Pathogenesis of Neuroblastoma	218	219	210	1.002210129	4.60752E-06	Disease Collections

Human LCL Control MPH vs. Saline

Name	# of Entities	Expanded # of Entities	# of Measured Entities	Median change	p-value	Hit type
nucleoplasm	2669	2669	2518	1.001257691	1.02974E-25	cellular_component
nucleus	6877	6877	5961	1.000046504	5.2368E-25	cellular_component
transcription, DNA-templated	2430	2430	2206	1.005042845	1.53769E-22	biological_process
regulation of transcription, DNA-templated	2670	2670	2279	1.00449709	4.42731E-22	biological_process
metal ion binding	3566	3568	3178	-1.000110916	2.42997E-18	molecular_function
DNA binding	2411	2411	2103	1.004503036	1.10098E-15	molecular_function
cytoplasm	6831	6831	6084	-1.000488189	1.54986E-15	cellular_component
positive regulation of transcription, DNA-templated	623	623	579	1.007308911	6.68324E-13	biological_process
nucleotide binding	1946	1946	1792	-1.000554726	1.42437E-12	molecular_function
nucleic acid binding	1094	1094	856	1.004358534	2.36262E-12	molecular_function
transcription factor activity, sequence-specific DNA binding	1125	1125	961	1.005918872	6.0621E-12	molecular_function
negative regulation of transcription from RNA polymerase II promoter	799	799	709	1.006048569	7.09537E-12	biological_process
positive regulation of transcription from RNA polymerase II promoter	1041	1041	947	1.006516349	2.51457E-11	biological_process
negative regulation of transcription, DNA-templated	590	590	506	1.006228895	3.27618E-11	biological_process

Human LCL aADHD vs. Control (both saline)

Name	# of Entities	Expanded # of Entities	# of Measured Entities	Median change	p-value	Hit type
cell junction	705	705	668	1.000670164	3.13357E-13	cellular_component
plasma membrane	5390	5390	3785	1.000481876	2.72858E-11	cellular_component
cytoplasm	6831	6831	6084	1.003976041	3.50727E-11	cellular_component
synapse	466	466	431	1.001119376	2.62418E-10	cellular_component
postsynaptic density	168	168	164	1.005543612	4.34769E-10	cellular_component
dendrite	396	396	373	-1.002282386	4.59111E-08	cellular_component
axon	318	318	304	1.000391067	4.45183E-07	cellular_component
neuron projection	378	378	357	1.00028352	5.35847E-07	cellular_component
axon guidance	385	385	374	1.000934601	1.1837E-06	biological_process
nervous system development	523	523	484	-1.000024763	1.71094E-06	biological_process
synaptic transmission	472	472	454	-1.001928998	1.79574E-06	biological_process
neuronal cell body	466	466	434	-1.002282386	4.89876E-06	cellular_component
dendritic spine	135	135	127	1.003774527	8.03048E-06	cellular_component

Human LCL aADHD MPH vs. Control MPH

Name	# of Entities	Expanded # of Entities	# of Measured Entities	Median change	p-value	Hit type
Secreted proteins	7555	12985	8259	1.001211728	2.11719E-18	Pathway Studio Ontology
plasma membrane	5390	5390	3785	1.004115727	1.3399E-16	cellular_component
signal transduction	1843	1843	1618	1.003692665	1.57657E-13	biological_process
membrane	7012	7012	6263	-1.000478468	3.59447E-12	cellular_component
immune response	468	468	369	-1.003564706	3.95518E-11	biological_process
Proteins Involved in Pathogenesis of Melanoma	245	264	240	1.00444264	6.62609E-11	Disease Collections
cell adhesion	616	616	564	1.006635379	8.16755E-11	biological_process
negative regulation of transcription from RNA polymerase II promoter	799	799	709	-1.000651379	9.04343E-11	biological_process
positive regulation of GTPase activity	494	494	446	1.002430828	1.14063E-10	biological_process

positive regulation of transcription from RNA polymerase II promoter	1041	1041	947	-1.000158886	1.53068E-10	biological_process
cell surface	645	645	573	1.004718042	3.43588E-10	cellular_component
Proteins Involved in Pathogenesis of Glioma	304	351	304	-1.001578048	5.99891E-10	Disease Collections
negative regulation of cell proliferation	471	471	433	-1.003522753	6.00697E-10	biological_process
Proteins Involved in Pathogenesis of Endometriosis	257	257	253	1.000672849	9.29616E-10	Disease Collections
axon guidance	385	385	374	1.007388538	1.8896E-06	biological_process
postsynaptic density	168	168	164	1.00902234	1.94917E-06	cellular_component

References

1. Sadasivan S, Pond BB, Pani AK, Qu C, Jiao Y, Smeyne RJ. Methylphenidate exposure induces dopamine neuron loss and activation of microglia in the basal ganglia of mice. *PLoS One* 2012; **7**(3)**:** e33693.

2. Dela Pena I, Jeon SJ, Lee E, Ryu JH, Shin CY, Noh M*, et al*. Neuronal development genes are key elements mediating the reinforcing effects of methamphetamine, amphetamine, and methylphenidate. *Psychopharmacology (Berl)* 2013; **230**(3)**:** 399-413.

3. Schwarz R, Reif A, Scholz CJ, Weissflog L, Schmidt B, Lesch KP*, et al*. A preliminary study on methylphenidate-regulated gene expression in lymphoblastoid cells of ADHD patients. *World J Biol Psychiatry* 2015; **16**(3)**:** 180-189.
